# Human Papillomavirus Vaccination for Adults: Updated Recommendations of the Advisory Committee on Immunization Practices

**DOI:** 10.15585/mmwr.mm6832a3

**Published:** 2019-08-16

**Authors:** Elissa Meites, Peter G. Szilagyi, Harrell W. Chesson, Elizabeth R. Unger, José R. Romero, Lauri E. Markowitz

**Affiliations:** ^1^National Center for Immunization and Respiratory Diseases, CDC; ^2^University of California at Los Angeles; ^3^National Center for HIV, Viral Hepatitis, STD, and TB Prevention, CDC; ^4^National Center for Emerging and Zoonotic Infectious Diseases, CDC; ^5^University of Arkansas for Medical Sciences, Little Rock, Arkansas.

## Introduction

Vaccination against human papillomavirus (HPV) is recommended to prevent new HPV infections and HPV-associated diseases, including some cancers. The Advisory Committee on Immunization Practices (ACIP)* routinely recommends HPV vaccination at age 11 or 12 years; vaccination can be given starting at age 9 years. Catch-up vaccination has been recommended since 2006 for females through age 26 years, and since 2011 for males through age 21 years and certain special populations through age 26 years. This report updates ACIP catch-up HPV vaccination recommendations and guidance published in 2014, 2015, and 2016 (*1*–*3*). Routine recommendations for vaccination of adolescents have not changed. In June 2019, ACIP recommended catch-up HPV vaccination for all persons through age 26 years. ACIP did not recommend catch-up vaccination for all adults aged 27 through 45 years, but recognized that some persons who are not adequately vaccinated might be at risk for new HPV infection and might benefit from vaccination in this age range; therefore, ACIP recommended shared clinical decision-making regarding potential HPV vaccination for these persons.

## Background

HPV is a common sexually transmitted infection, with HPV acquisition generally occurring soon after first sexual activity (*1*). Most HPV infections are transient and asymptomatic. Persistent infections with high-risk (oncogenic) HPV types can lead to development of cervical, anal, penile, vaginal, vulvar, and oropharyngeal cancers, usually after several decades (*1*). Most new HPV infections occur in adolescents and young adults. Although most sexually active adults have been exposed to HPV (*4*), new infections can occur with a new sex partner (*5*).

Three prophylactic HPV vaccines are licensed for use in the United States: 9-valent (9vHPV, Gardasil 9, Merck), quadrivalent (4vHPV, Gardasil, Merck), and bivalent (2vHPV, Cervarix, GlaxoSmithKline) (*6*–*8*). As of late 2016, only 9vHPV is distributed in the United States. The majority of HPV-associated cancers are caused by HPV 16 or 18, types targeted by all three vaccines. In addition, 4vHPV and 9vHPV target HPV 6 and 11, types that cause anogenital warts. 9vHPV also protects against five additional high-risk types: HPV 31, 33, 45, 52, and 58.

In October 2018, using results from 4vHPV clinical trials in women aged 24 through 45 years, and bridging immunogenicity and safety data in women and men, the Food and Drug Administration expanded the approved age range for 9vHPV use from 9 through 26 years to 9 through 45 years in women and men (*6*). In June 2019, after reviewing evidence related to HPV vaccination of adults, ACIP updated recommendations for catch-up vaccination and for vaccination of adults older than the recommended catch-up age.

## Methods

During April 2018–June 2019, the ACIP HPV Vaccines Work Group held at least monthly conference calls to review and discuss relevant scientific evidence regarding adult HPV vaccination using the Evidence to Recommendations framework. (https://www.cdc.gov/vaccines/acip/recs/grade/downloads/ACIP-evidence-rec-frame-508.pdf). The Work Group evaluated the quality of evidence for efficacy, safety, and effectiveness for HPV vaccination for primary prevention of HPV infection and HPV-related disease using the Grading of Recommendations Assessment, Development and Evaluation (GRADE) approach (https://www.cdc.gov/vaccines/acip/recs/grade/about-grade.html).

Scientific literature published during January 1, 2006–October 18, 2018, was searched to identify clinical trials of any licensed HPV vaccine in adults aged 27 through 45 years. Detailed search methods and results for the GRADE tables are available at https://www.cdc.gov/vaccines/acip/recs/grade/HPV-adults.html. Benefits were based on per-protocol analyses of vaccine efficacy; immunogenicity data were also considered. Harms were any vaccine-related serious adverse events. Of 1,388 references identified, 100 were selected for detailed review, and 16 publications were included in GRADE tables presented at the October 2018 ACIP meeting; tables were updated in June 2019 to include new results from a 9vHPV trial. At the June 2019 ACIP meeting, two policy issues were considered: 1) harmonization of catch-up vaccination for all persons through age 26 years, and 2) vaccination of adults aged >26 years. Two Evidence to Recommendations documents were developed (https://www.cdc.gov/vaccines/acip/recs/grade/HPV-harmonization-etr.html) (https://www.cdc.gov/vaccines/acip/recs/grade/HPV-adults-etr.html) and presented along with proposed recommendations; after a public comment period, ACIP members voted unanimously to harmonize catch-up vaccination recommendations across genders for all persons through age 26 years. ACIP members also voted 10–4 in favor of shared clinical decision-making for adults aged 27 through 45 years, recognizing that some persons who are not adequately vaccinated might be at risk for new HPV infection and might benefit from vaccination in this age range.

## Summary of Key Findings

**Vaccine efficacy and safety.** Data were considered from 11 clinical trials of 9vHPV, 4vHPV, and/or 2vHPV in adults aged 27 through 45 years, along with supplemental bridging immunogenicity data. In per-protocol analyses from three trials, 4vHPV and 2vHPV demonstrated significant efficacy against a combined endpoint of persistent vaccine-type HPV infections, anogenital warts, and cervical intraepithelial neoplasia (CIN) grade 1 (low-grade lesions) or worse. In nine trials, seroconversion rates to vaccine-type HPV after 3 doses of any HPV vaccine were 93.6%–100% at 7 months after the first dose. Overall evidence on benefits was GRADE evidence level 2, for moderate-quality evidence. In nine trials, few serious adverse events and no vaccine-related deaths were reported. Overall evidence on harms was also GRADE evidence level 2, for moderate-quality evidence. In the efficacy trial that was the basis for 9vHPV licensure for adults through age 45 years, per-protocol efficacy of 4vHPV among women aged 24 through 45 years was 88.7% (95% confidence interval [CI] = 78.1–94.8), and intention-to-treat efficacy was 47.2% (95% CI = 33.5–58.2) against a combined endpoint of persistent infections, extragenital lesions, and CIN 1+ related to HPV types 6, 11, 16, or 18 (*9*).

**HPV burden of disease and impact of the vaccination program in the United States.** Approximately 33,700 cancers are caused by HPV in the United States each year, including 12,900 oropharyngeal cancers among men and women, 10,800 cervical cancers among women, and 6,000 anal cancers among men and women; vaginal, vulvar, and penile cancers are less common (*10*). HPV vaccination for adolescents has been routinely recommended for females since 2006 and for males since 2011 (*1*). The existing HPV vaccination program for adolescents has the potential to prevent the majority of these cancers. Mean age at acquisition of causal HPV infection for cancers is unknown, but is estimated to be decades before cancer is diagnosed. In 2017, coverage with ≥1 dose of HPV vaccine was 65.5% among adolescents aged 13 through 17 years (*11*). Although coverage with the recommended number of doses remains below the Healthy People 2020 target of 80% for adolescents (*12*), the U.S. HPV vaccination program has resulted in significant declines in prevalences of vaccine-type HPV infections, anogenital warts, and cervical precancers (*13*). For example, prevalences of 4vHPV vaccine-type infection during 2013–2016, compared with those of the prevaccine era, declined from 11.5% to 1.8% among females aged 14 through 19 years and from 18.5% to 5.3% among females aged 20 through 24 years (*14*). In addition, declines have been observed among unvaccinated persons, suggesting protective herd effects (*15*).

**Health economic analyses.** Five health economic models of HPV vaccination in the United States were reviewed (*16*). The cost effectiveness ratio for the current HPV vaccination program ranged from cost-saving to approximately $35,000 per quality-adjusted life year (QALY) gained (*16*). In the context of the existing vaccination program, the incremental cost per QALY for expanding male vaccination through age 26 years was $178,000 in a subset of analyses in one of the five models reviewed using more favorable model assumptions for adult vaccination (*16*). In the context of the existing program, expanding vaccination to adults through age 45 years would produce relatively small additional health benefits and less favorable cost-effectiveness ratios. The incremental cost per QALY for also vaccinating adults through age 30 or 45 years exceeded $300,000 in four of five models (*16*). Variation in results across models was likely due to uncertainties about HPV natural history, such as prevalence of immunity after clearance of natural infections, and level of herd protection from the existing program. Under the existing program, in a subset of analyses in one of the five models reviewed using more favorable model assumptions for adult vaccination, the number needed to vaccinate (NNV) to prevent one case of anogenital warts, CIN grade 2 or worse (high-grade lesions), or cancer would be 9, 22, and 202, respectively. For expanding recommendations for males through age 26 years to harmonize catch-up vaccination across genders, these NNV would be 40, 450, and 3,260, respectively. For expanding recommendations to include adults through age 45 years, these NNV would be 120, 800, and 6,500, respectively (*16*).

## Rationale

Adolescents remain the most important focus of the HPV vaccination program in the United States. Recommendations harmonized across genders will simplify the immunization schedule and be more feasible to implement. HPV vaccination is most effective when given before exposure to any HPV, as in early adolescence (*1*–*3*). Clinical trials have indicated that HPV vaccines are safe and effective against infection and disease attributable to HPV vaccine types that recipients are not infected with at the time of vaccination.

Because HPV acquisition generally occurs soon after first sexual activity, vaccine effectiveness will be lower in older age groups because of prior infections. Some previously exposed adults will have developed natural immunity already. Exposure to HPV decreases among older age groups. Evidence suggests that although HPV vaccination is safe for adults aged 27 through 45 years, population benefit would be minimal; nevertheless, some adults who are not adequately vaccinated might be at risk for new HPV infection and might benefit from vaccination in this age range.

## Recommendations

**Children and adults aged 9 through 26 years.** HPV vaccination is routinely recommended at age 11 or 12 years; vaccination can be given starting at age 9 years. Catch-up HPV vaccination is recommended for all persons through age 26 years who are not adequately vaccinated.^†^

**Adults aged >26 years.** Catch-up HPV vaccination is not recommended for all adults aged >26 years. Instead, shared clinical decision-making regarding HPV vaccination is recommended for some adults aged 27 through 45 years who are not adequately vaccinated. (Box). HPV vaccines are not licensed for use in adults aged >45 years.

BOX **Considerations for shared clinical decision-making regarding human papillomavirus (HPV) vaccination of adults aged 27 through 45 years**

Ideally, HPV vaccination should be given in early adolescence because vaccination is most effective before exposure to HPV through sexual activity. For adults aged 27 through 45 years who are not adequately vaccinated,* clinicians can consider discussing HPV vaccination with persons who are most likely to benefit. HPV vaccination does not need to be discussed with most adults aged >26 years.

- HPV is a very common sexually transmitted infection. Most HPV infections are transient and asymptomatic and cause no clinical problems.
- Although new HPV infections are most commonly acquired in adolescence and young adulthood, some adults are at risk for acquiring new HPV infections. At any age, having a new sex partner is a risk factor for acquiring a new HPV infection.
- Persons who are in a long-term, mutually monogamous sexual partnership are not likely to acquire a new HPV infection.
- Most sexually active adults have been exposed to some HPV types, although not necessarily all of the HPV types targeted by vaccination.
- No clinical antibody test can determine whether a person is already immune or still susceptible to any given HPV type.
- HPV vaccine efficacy is high among persons who have not been exposed to vaccine-type HPV before vaccination.
- Vaccine effectiveness might be low among persons with risk factors for HPV infection or disease (e.g., adults with multiple lifetime sex partners and likely previous infection with vaccine-type HPV), as well as among persons with certain immunocompromising conditions.
- HPV vaccines are prophylactic (i.e., they prevent new HPV infections). They do not prevent progression of HPV infection to disease, decrease time to clearance of HPV infection, or treat HPV-related disease.

* Dosing schedules, intervals, and definitions of persons considered adequately vaccinated have not changed.

**Administration. **Dosing schedules, intervals, and definitions of persons considered adequately vaccinated have not changed (*3*). No prevaccination testing (e.g., Pap or HPV testing) is recommended to establish the appropriateness of HPV vaccination. 

**Cervical cancer screening.** Cervical cancer screening guidelines and recommendations should be followed (*17*).

**Special populations and medical conditions.** These recommendations for children and adults aged 9 through 26 years and for adults aged >26 years apply to all persons, regardless of behavioral or medical risk factors for HPV infection or disease.^§^ For persons who are pregnant, HPV vaccination should be delayed until after pregnancy; however, pregnancy testing is not needed before vaccination. Persons who are breastfeeding or lactating can receive HPV vaccine. Recommendations regarding HPV vaccination during pregnancy or lactation have not changed (*1*).

## Future Research and Monitoring Priorities

CDC continues to monitor safety of HPV vaccines and impact of the vaccination program on HPV-attributable outcomes, including prevalences of HPV infections, anogenital warts, cervical precancers, and cancers. ACIP reviews relevant data as they become available and updates vaccine policy as needed.

**Summary**

**What is already known about this topic?**

Vaccination against human papillomavirus (HPV) is routinely recommended at age 11 or 12 years. Catch-up recommendations apply to persons not vaccinated at age 11 or 12 years.

**What is added by this report?**

After reviewing new evidence, CDC updated HPV vaccination recommendations for U.S. adults.

**What are the implications for public health practice?**

Routine recommendations for HPV vaccination of adolescents have not changed. Catch-up HPV vaccination is now recommended for all persons through age 26 years. For adults aged 27 through 45 years, public health benefit of HPV vaccination in this age range is minimal; shared clinical decision-making is recommended because some persons who are not adequately vaccinated might benefit.

## Footnotes

* Recommendations for routine use of vaccines in children, adolescents, and adults are developed by the Advisory Committee on Immunization Practices (ACIP). ACIP is chartered as a federal advisory committee to provide expert external advice and guidance to the Director of CDC on use of vaccines and related agents for the control of vaccine-preventable diseases in the civilian population of the United States. Recommendations for routine use of vaccines in children, adolescents, and adults are harmonized to the greatest extent possible with recommendations made by the American Academy of Pediatrics (AAP), the American Academy of Family Physicians (AAFP), and the American College of Obstetricians and Gynecologists (ACOG). Recommendations for routine use of vaccines in adults are harmonized with recommendations of AAFP, ACOG, the American College of Physicians (ACP), and the American College of Nurse-Midwives. ACIP recommendations approved by the CDC Director become agency guidelines on the date published in the *Morbidity and Mortality Weekly Report*. Additional information is available at https://www.cdc.gov/vaccines/acip.

† For persons initiating vaccination before their 15th birthday, the recommended immunization schedule is 2 doses of HPV vaccine (0, 6–12 month schedule). For persons initiating vaccination on or after their 15th birthday, or for persons with certain immunocompromising conditions, the recommended immunization schedule is 3 doses of HPV vaccine (0, 1–2, 6 month schedule).

§ Persons with specific behavioral or medical risk factors for HPV infection or disease include men who have sex with men, transgender persons, and persons with immunocompromising conditions.
